# Prosociality and hoarding amid the COVID‐19 pandemic: A tale of four countries

**DOI:** 10.1002/casp.2516

**Published:** 2021-04-05

**Authors:** Dwight C. K. Tse, Vienne W. Lau, Ying‐yi Hong, Michelle C. Bligh, Maria Kakarika

**Affiliations:** ^1^ School of Psychological Sciences and Health University of Strathclyde Glasgow UK; ^2^ Department of Psychology The Chinese University of Hong Kong Hong Kong Hong Kong; ^3^ Department of Management University of Central Oklahoma Edmond Oklahoma USA; ^4^ Department of Marketing The Chinese University of Hong Kong Hong Kong Hong Kong; ^5^ Division of Behavioral and Organizational Sciences Claremont Graduate University Claremont California USA; ^6^ EM Normandie Business School Métis Lab Paris France

**Keywords:** donation, moral identity, panic buying, psychological well‐being, stockpiling, stress and coping

## Abstract

The COVID‐19 pandemic is an unprecedented public health crisis that poses a challenge to humanity. Drawing on the stress and coping literature, we argue that people around the world alleviate their anxiety and stress induced by the pandemic through both prosocial and ‘self‐interested’ hoarding behaviours. This cross‐cultural survey study examined the pushing (threat perception) and pulling (moral identity) factors that predicted prosocial acts and hoarding, and subsequently psychological well‐being. Data were collected from 9 April to 14 May 2020 from 251 participants in the United Kingdom (UK), 268 in the United States (US), 197 in Germany (DE), and 200 in Hong Kong (HK). Whereas threat perception was associated positively with both prosocial acts and hoarding, benevolent moral identity was associated positively with the former but not the latter behaviour. We also observed cross‐cultural differences, such that both effects were stronger in more individualistic (UK, US) countries than less individualistic (HK, DE) ones. The findings shed light on the prosocial vs. self‐interested behavioural responses of people in different cultures towards the same pandemic crisis.

## 1 INTRODUCTION

The COVID‐19 pandemic is a public health crisis that has brought immense psychological stress to people around the globe (Chew et al., 2020; Wang et al., 2020; Xiong et al., 2020). Aside from primary stress associated with the fear of infections, people also suffer from secondary stress, or adversity associated with the pandemic, including shortage of supplies, unemployment, disruptions of daily routines, and lack of access to psychiatric care (e.g., Hao et al., 2020). As such, psychologists and public health professionals have warned against the decline in psychological well‐being, such as lower feelings of success in life (i.e., flourishing) and negative affective balance, resulting from the pandemic (e.g., Birditt, Turkelson, Fingerman, Polenick, & Oya, 2020).

During times of crisis, people seek to alleviate the stress and anxiety induced by the stressor. Their observed coping responses during the pandemic vary from heroic and prosocial acts to accentuated self‐interested behaviours. For example, at the onset of the pandemic, people across the world reportedly donated supplies to others in need but also emptied the shelves in grocery stores, despite government officials denying the urgency of stocking up on food and essential items. Although engaging in both prosocial and seemingly self‐interested acts during the pandemic appears puzzling at first, psychological scholars understand a wide range of such behaviours as a form of the stress response (e.g., Arafat, Kar, & Kabir, 2020; Ho, Chee, & Ho, 2020). Nonetheless, the majority of news reports have attributed hoarding behaviour – the over‐acquisition, accumulation, and unwillingness of discarding a large number of limited value items (Kress, Stargell, Zoldan, & Paylo, 2016), to the power of rumours and blamed people as ‘irrational’ (Arafat et al., 2020). Such an oversimplified view of the crowd as selfish or ‘irrational’ may result in overlooking underlying factors that explain hoarding and prosocial behaviour during the pandemic.

The observation of both prosocial and seemingly self‐interested acts during the pandemic appears puzzling at first. In this study, we sought to explain prosociality and hoarding through insights derived from the stress and coping model. We tested the idea that people both hoard food and sanitary items, and share supplies with others, to cope with the stress induced by the COVID‐19 crisis. While prosociality takes many forms in usual circumstances (e.g., volunteerism, caregiving), we limited our scope to donation of supplies to and sharing information with others, given that many forms of prosocial acts are restricted or discouraged due to social distancing and lockdown measures. We also investigated how moral identity further distinguishes people who are prone to act prosocially or in a self‐interested way from others. Finally, we evaluated the impact of prosociality and hoarding on psychological well‐being. Given the COVID‐19 pandemic as a global phenomenon, we also studied whether people in different cultures behave similarly. Our findings are discussed in terms of implications for both research on crises and practice in the fields of public health administration and consumer behaviour.

### 1.1 Coping with COVID‐19: Prosociality and hoarding

According to the stress and coping framework (Lazarus & Folkman, 1984), people perceive stress when they evaluate that the environmental demands exceed the resources they possess. The process of cognitive appraisal begins with the evaluation of whether the situation (a potential stressor) threatens to harm a person's well‐being (primary appraisal), followed by the evaluation of whether a person has sufficient resources to deal with the stressor (secondary appraisal). This appraisal process leads to the employment of different coping strategies and, ultimately, to adaptation or maladaptation. Besides the original stress and coping model, the organisational psychology literature on how people cope with natural disasters (e.g., hurricanes and earthquakes) has recognised the importance of collective stress – a sense of insufficient collective resources in comparison to the threat induced by a shared environment – in determining the coping strategies adopted and the relative success in adaption (e.g., Jonas, 2012; Rodríguez, Trainor, & Quarantelli, 2006). Similar to other catastrophes in history, the COVID‐19 pandemic can be stressful to individual persons and collectively to citizens in society (Lansisalmi, Peiro, & Kivimaki, 2000).

In the context of the COVID‐19 pandemic, the more people perceive the pandemic as a threat because they or their communities do not have enough resources to survive it, the more likely they are to adjust their behaviour to cope with the threat. The COVID‐related threat is manifested in many forms, such as a potentially life‐threatening infection, shortage of essential items due to disruption of production and supply chains, and the inability to fulfil basic needs due to city‐ or country‐wide lockdown. Because the pandemic is a stressor at both personal and community levels, taking actions to ensure adequate supplies and survive the lockdown can be considered as problem‐focused coping strategies, or the effort of ‘solving or managing the problem that is causing the distress’ (Folkman & Moskowitz, 2000, p. 650). Among these strategies, whereas stockpiling food and sanitary/hygienic products is an attempt to deal with the personal shortage of supplies, donation and giving out tangible resources is an attempt to handle the community shortage of supplies.

Previous studies have supported the notion that threat perception is associated with both altruistic and ‘self‐interested’ behaviour (e.g., Li, Song, & Xie, 2020). In other society‐wide crises such as Hurricane Katrina in the U.S., prosocial and hoarding behaviours were observed simultaneously as people's lives were under threat (Rodríguez et al., 2006). Although hoarding is not ‘antisocial’ by its nature, stockpiling excess items while there is shortage of supplies is likely to lead to others' inability to obtain the supplies. Therefore, donation and hoarding can be considered as contrasting behaviours that nevertheless can occur simultaneously. We thus hypothesise that both behaviours will be directly predicted by the levels of perceived threat.

*The more people perceive COVID‐19 as a threat, the more likely they engage in both prosocial acts (H1a) and hoarding behaviour (H1b)*.

### 1.2 Moral identity, prosociality, and hoarding

In parallel with threat perception (a situational factor) that may ‘push’ people towards higher anxiety during the COVID‐19 pandemic, there are also dispositional factors that may ‘pull’ people from anxiety and influence prosocial acts and hoarding in response to a shortage of supplies. In this study, we focus on moral identity – the self‐conception associated with a set of moral dispositions, such as benevolence, justice, obligation, and integrity (Hannah, Thompson, & Herbst, 2018). The more strongly people identify themselves as benevolent, the greater tendency they have to adhere to the benevolence principles and act prosocially, such as volunteering, donations, and organisational citizenship behaviour (Colby & Damon, 1992; Winterich, Aquino, Mittal, & Swartz, 2013). In contrast, a stronger moral identity should discourage self‐interested behaviour such as hoarding, which ignores others' interests amid a shortage of supplies. The main effect of moral identity on prosociality is consistent across contexts, even when resources are scarce (Reed & Aquino, 2003). As such, we posit that during the pandemic, benevolent moral identity should encourage donation and expressing care to others in need and discourage stockpiling essential items.

*Identification with benevolent moral identity is positively associated with prosocial acts (H2a) and negatively associated with hoarding (H2b) during the COVID‐19 pandemic*.

### 1.3 Effects of prosociality and hoarding on psychological well‐being

While both donating and hoarding, as problem‐focused coping strategies, should engender a sense of control over the stressor (Lazarus & Folkman, 1984; Moskowitz, Folkman, Collette, & Vittinghoff, 1996), we posit that prosocial acts (vs. hoarding supplies) can better help people cope with the COVID‐19‐induced stress. Although hoarding guarantees a wealth of personal resources, it collectively exacerbates the shortage of supplies at the community level. This coping strategy is thus likely to be maladaptive (or at least, less adaptive) and ultimately related to poorer well‐being. Conversely, donation and sharing items does not only imply an abundance of personal resources, but it also mitigates the panic of insufficient resources in the community (Arafat, Kar, & Kabir, 2020; Bekkers & Wiepking, 2007). This coping strategy thus addresses the shortage problem at both personal and community levels and is likely to enhance psychological well‐being. In the literature, there is ample evidence that prosociality is associated with better psychological well‐being (e.g., Dunn, Aknin, & Norton, 2008; Hui et al., 2020), whereas hoarding is related to poorer well‐being (e.g., Prentice, Quach, & Thaichon, 2020; Tolin et al., 2019). Therefore, although both coping strategies may have relieve COVID‐related stress, compared to hoarding, engagement in prosocial acts should have a stronger, positive association with psychological well‐being.

*Compared to hoarding, prosocial behaviour has a stronger positive association with psychological well‐being during the COVID‐19 pandemic*.

### 1.4 The role of culture in behavioural responses

During the COVID‐19 outbreak, whereas some countries have exported medical supplies to countries in need, others have shown hesitation to share and instead stockpiled drugs and essential items (BBC, 2020a; Blanchard, 2020). Cultural differences in response to the crisis can be observed not only at the national level but also at the individual level. For example, at the onset of the pandemic, people in different cultures had opposing opinions regarding the use of face masks – one of the most hoarded items (Wang et al., 2020). We thus argue that cultural differences will moderate how threat perception and moral identity influence prosocial acts and hoarding.

First, cultural dimensions such as individualism/collectivism may determine to what extent people perceive COVID‐19 as a collective stressor to the community. Whereas self‐focused, ‘me’‐oriented thinking is promoted in individualistic cultures, other‐focused, ‘we’‐oriented thinking is celebrated in collectivistic cultures (Chentsova‐Dutton & Tsai, 2010). Accordingly, people in individualistic cultures are likely to perceive COVID‐19 as a personal crisis, while those in collectivistic cultures are likely to perceive it as a collective crisis. While coping with the pandemic, people in individualistic cultures are likely to hoard rather than donate and share essential items. Conversely, people in collectivistic cultures are likely to act prosocially by donate food and supplies to people in need.

This assertion is consistent with the stronger emphasis on social harmony in collectivistic than individualistic cultures, especially when there is potential conflict between personal welfare and the welfare of others (Triandis, 1995). People in collectivistic (vs. individualistic) cultures are likely to conform to the social responsibility of taking care of others in need (Finkelstein, 2011). In the organisational behaviour literature, organisational citizenship behaviour is associated positively with collective identity but negatively with individualistic identity (Ramarajan, Berger, & Greenspan, 2017), suggesting that collectivistic thinking promotes prosociality. Therefore, whereas perceiving COVID‐19 as a threat should have a stronger effect on hoarding in individualistic than collectivistic cultures, it should also have a stronger effect on prosocial acts in collectivistic than individualistic cultures:

*The positive relationship between the COVID‐19 threat perception and prosocial acts is stronger in collectivistic than in individualistic cultures*.

*The positive relationship between the COVID‐19 threat perception and hoarding is stronger in individualistic than in collectivistic cultures*.

Second, given that people in individualistic cultures have a stronger tendency to act in accordance with their self‐concepts, prosocial acts may be more closely linked to moral identity in individualistic cultures (Henrich et al., 2001). In collectivistic cultures, people's (prosocial) behaviour is more motivated by external factors such as social norm adherence, conformity, and social harmony (Jia & Krettenauer, 2017; Markus & Kitayama, 1991). As such, the unique effect of moral identity on determining prosocial acts during the COVID‐19 pandemic may be weaker in collectivistic cultures. Indeed, a meta‐analysis on moral identity and moral behaviour has revealed its relationship being stronger in individualistic than collectivistic cultures (Hertz & Krettenauer, 2016). With a similar reasoning, moral identity will reduce hoarding behaviours more strongly in individualistic rather than in collectivistic cultures. Therefore,

*The positive relationship between benevolent moral identity and prosocial acts during the COVID‐19 pandemic is stronger in individualistic cultures than in collectivistic cultures*.

*The negative relationship between benevolent moral identity and hoarding during the COVID‐19 pandemic is stronger in individualistic cultures than in collectivistic cultures*.

## 2 THE CURRENT STUDY

**FIGURE 1:**
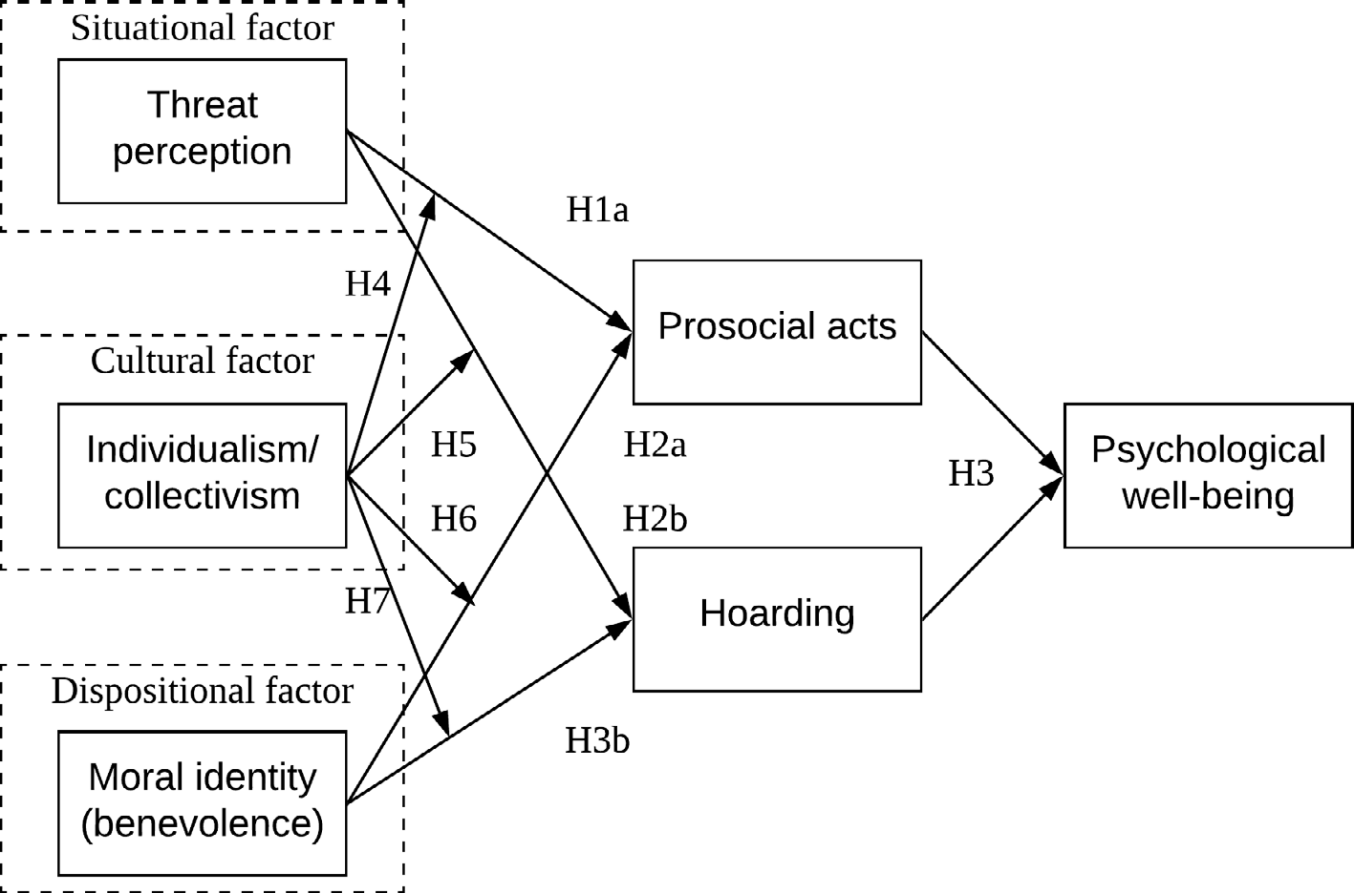
A conceptual model and hypotheses ([Link], [Link], [Link], [Link], [Link], [Link], [Link])

We tested our hypotheses by conducting a cross‐cultural survey study. We collected data on both prosocial and ‘self‐interested’, hoarding behaviours at the onset of the COVID‐19 pandemic, in four sites across the world. We examined whether situational (threat perception) and dispositional (moral identity) factors predicted prosocial and hoarding acts in different cultures, and how they were subsequently related to psychological well‐being. Our hypothesised model is depicted in Figure 1.

## 3 METHOD

### 3.1 Participants

We recruited participants aged 18 or above who read English proficiently from various online platforms, namely Consumer Fieldwork in the U.K. (*n* = 251) and Germany (*n* = 197), Amazon MTurk in the U.S. (*n* = 268), and Qualtrics panel in Hong Kong (*n* = 200). These countries represent the wide individualistic–collectivistic spectrum, with the U.K. and the U.S. being high in individualism, followed by Germany and Hong Kong (Hofstede, 1980). Based on power analysis results (Faul, Erdfelder, Buchner, & Lang, 2009), we recruited at least 152 participants from each location to detect partial *R* ^2^ = .05 with alpha = .05 and power = .80 in linear regression and path analyses. We decided to oversample 30% to ensure stable coefficient estimates and allow room to eliminate low‐quality data. We excluded 59 participants due to poor‐quality responses and univariate outliers. Table 1 shows the descriptive statistics for each subsample.

**TABLE 1:** Descriptive statistics of variables in the U.S., the U.K., Germany, and Hong Kong

Variables	*M*(*SD*)/%				Omnibus tests (*F*/*χ* ^2^)
	U.K.	U.S.	Germany	Hong Kong	
Age	58.20 (14.44)	40.56 (12.79)	48.16 (13.43)	42.44 (13.63)	84.72***
Gender (% female)	48.39	51.52	50.51	51.26	0.59
Subjective SES (1–10)	5.47 (1.61)	4.79 (1.68)	5.99 (1.61)	5.65 (1.50)	23.32***
Education (1–8)	3.57 (1.67)	4.43 (1.21)	5.10 (1.40)	4.41(1.58)	40.60***
Liberal (1–7)	3.60 (1.64)	4.09 (2.34)	4.55 (1.35)	4.78 (1.42)	19.26***
Conservative (1–7)	3.91 (1.87)	3.79 (2.35)	3.51 (1.52)	3.70 (1.43)	1.78
Flourishing (1–7)	4.86 (1.14)	4.97 (1.26)	4.81 (1.17)	4.63 (0.94)	3.39*
Affect (1–7)	4.49 (1.05)	4.35 (1.17)	4.34 (0.97)	3.88 (0.81)	14.26***
Prosocial acts (1–7)	4.17 (1.57)	4.38 (1.64)	3.93 (1.31)	4.08 (1.12)	3.94**
Hoarding (1–7)	3.26 (1.73)	4.02 (1.96)	2.52 (1.45)	3.83 (1.61)	33.43***
Threat perception (1–5)	3.89 (0.77)	3.89 (0.84)	3.38 (0.64)	3.91 (0.67)	24.53***
Moral identity (1–5)					
Benevolence	6.01 (1.02)	5.69 (1.33)	5.13 (1.30)	5.13 (0.97)	31.58***
Justice	5.85 (0.93)	5.67 (1.12)	5.09 (1.19)	4.92 (0.94)	40.54***
Obligation	6.32 (0.79)	6.06 (1.07)	5.38 (1.23)	5.16 (0.95)	66.53***
Integrity	6.41 (0.73)	6.04 (1.04)	5.53 (1.27)	5.30 (0.97)	56.14***

*Note: n*_UK_ = 251. *n*_US_ = 268. *n*_DE_ = 197. *n*_HK_ = 200. SES = socioeconomic status. For gender, we only included binary genders.

^*^ *p* < .05.

^**^ *p* < .01.

^***^ *p* < .001.

### 3.2 Procedure

Participants completed an online survey in English. Due to the unanticipated fast development of the COVID‐19 pandemic and our desire to launch the surveys in closer time to increase comparability across countries, we only developed an English version of the survey and set proficient English‐reading as an inclusion criterion. We launched the survey from 9 April to 14 May 2020, while the global confirmed infections rose from 1,505,137 to 4,298,956. The study had obtained ethics approval from universities in each location. Because all materials were hosted online, participation did not inflict additional risks of COVID‐19 infections.

### 3.3 Measures

#### 3.3.1 Threat perception

Adapting from previous studies on the public health crisis associated with the severe acute respiratory syndrome (SARS; e.g., Vartti et al., 2009), we created a 6‐point threat perception scale to measure the perception of COVID‐19 as a threat. Participants rated each statement on a 5‐point Likert scale from 1 (*not at all*) to 5 (*a great deal*). A sample item was ‘to what extent do you worry that COVID‐19 would harm the economy in [corresponding countries]’. The items were similar to those being used in another COVID‐19 behavioural study (Oosterhoff & Palmer, 2020). We exploratory factor analyses (EFA) with principal axis factoring and oblimin rotation, which suggested a one‐factor structure, with 44.76% variances extracted. Loadings ranged from .472 to .845 (see Table S1). We computed the composite scores by averaging the items. The Cronbach's alpha was .821.

#### 3.3.2 Moral identity

We used the moral identity questionnaire developed by Hannah et al. (2018). Participants rated to what extent the 12 attributes described them on a 7‐point Likert scale from 1 (*not at all like me*) to 7 (*very much like me*). These attributes covered four dimensions of moral identity, namely benevolence (e.g., caring), justice (e.g., fair), obligation (e.g., dependable), and integrity (e.g., honest). Whereas the benevolence dimension was our research focus, we also included the rest of the dimensions as covariates in subsequent analyses (see Analysis Plan). We created the composite scores by averaging the corresponding items. The Cronbach's alphas were .912 (benevolence), .835 (justice), .859 (obligation), and .899 (integrity).

#### 3.3.3 Prosocial acts and hoarding

Based on the conceptualisation of prosocial acts and self‐interested, hoarding behaviour in the literature (e.g., Hirschberger, Ein‐Dor, & Almakias, 2008; Twenge, Baumeister, DeWall, Ciarocco, & Bartels, 2007), we developed nine question items to measure participants' likelihood of engaging in various behaviours during the COVID‐19 pandemic on a 7‐point Likert scale from 1 (*extremely unlikely*) to 7 (*extremely likely*). Whereas an example of prosocial acts (six items) was ‘donating supplies to the needy’, an example of hoarding (three items) was ‘stocking up on toilet paper’. The items were similar to those being used in another COVID‐19 behavioural study (Oosterhoff & Palmer, 2020). We conducted EFA with principal axis factoring and oblimin rotation, which suggested a two‐factor structure, with 48.05% variances extracted by the first factor (prosocial acts) and 24.13% extracted by the second (hoarding behaviour). Loadings ranged from .467 to .961 (see Table S1). We computed the composite scores by averaging the corresponding items. The Cronbach's alphas of prosocial acts and hoarding were .908 and .932, respectively.

#### 3.3.4 Psychological well‐being

We operationalised psychological well‐being with the 8‐item flourishing scale and the 12‐item scale of positive and negative experience (SPANE) developed by Diener et al. (2009), similar to other studies (e.g., Demerouti, Bakker, & Gevers, 2015; Sandstrom & Dunn, 2014). For the flourishing scale, participants rated the extent to which they agreed with statements about their life recently (e.g., ‘Lately, I lead a purposeful and meaningful life’.) on a 7‐point scale from 1 (*strongly disagree*) to 7 (*strongly agree*). For SPANE, participants reported the frequency of experiencing six positive (e.g., pleasant, happy) and six negative (e.g., unpleasant, sad) affective states during the past month on a 7‐point Likert scale from 1 (*never*) to 7 (*always*). We created the composite scores by averaging the corresponding items (with negative affect reverse‐coded beforehand). The Cronbach's alphas of flourishing and affective balance were .911 and .921, respectively.

#### 3.3.5 Demographics

We also controlled for the variables that are likely to influence threat perceptions and subsequent behaviours. Specifically, we collected participants' age, gender, subjective socioeconomic status (1 = lowest status, 10 = highest status), education level, and their tendencies to endorse liberal and conservative ideologies from 1 (*strongly disagree*) to 7 (*strongly agree*; separate items).

### 3.4 Analysis plan

We first conducted confirmatory factor analyses (CFA) to make sure that all scale items loaded significantly to their corresponding factor. We evaluated acceptable model fit with CFI > .90, RMSEA < .06, and SRMR < .08 (Kline, 2015). We also evaluated the metric invariance of the items by comparing models with or without the factor loadings constrained to be equal across countries. Minimal differences in fit indices (ΔCFI < .01 and ΔRMSEA < .05) between these models indicated that factor loadings did not significantly differ across countries (Cheung & Rensvold, 2002).

For hypothesis testing, we conducted indirect effect analyses with the PROCESS macro for SPSS (Hayes, 2013), with parameter estimations based on 5,000 bootstrapped samples. We modelled threat perception and moral identity as the predictors, prosocial acts and hoarding as the parallel mediators, and psychological well‐being (operationalised as flourishing and affective balance) as the outcome. All demographic variables and other dimensions of moral identity (justice, obligation, integrity) were included as covariates. We first estimated moderated indirect effect models with all regression paths moderated by culture. None of the moderations of culture on threat perception/moral identity–well‐being outcomes (c′ paths) and those on prosocial acts/hoarding–well‐being outcomes (b paths) was statistically significant (see Table S3). We thus estimated another set of models that only included the interaction between culture and threat perception/moral identity on prosocial acts/hoarding (a paths) consistent with our hypotheses.

## 4 RESULTS

The data of this study are openly available in the Open Science Framework at https://osf.io/qf42h. In preliminary analyses, the model fit of the CFA of all items was acceptable, *χ*2(609) = 2076, *p* < .001, CFI = .93, RMSEA = .06, 90% CI [.05, .06], SRMR = .07. Standardised factor loadings ranged from .48 to .95. In addition, the model constraining factor loadings to be equal across countries had a similar model fit ΔCFI = .005, ΔRMSEA = .001, ΔSRMR = .006, suggesting good metric invariance of the items. Table S4 is the correlational matrix of the variables of interest. First, the findings supported H1a and H1b: greater threat perception was associated with more prosocial acts (*b* = 0.39, 95% CI [0.25, 0.53]) and hoarding (*b* = 0.50, 95% CI[0.34, 0.66]).

Second, stronger benevolent moral identity was associated with more prosocial acts (*b* = 0.61, 95% CI [0.51, 0.70]). However, it was not significantly related to hoarding behaviour (*b* = −0.00, 95% CI [−0.18, 0.18]). The findings supported H2a but not H2b, such that benevolent moral identity was only positively associated with prosocial acts but not with hoarding.

Third, an increased likelihood of prosocial acts was associated with better flourishing (*b* = 0.18, 95% CI [0.13, 0.23]). This finding supported H3. Surprisingly, while we originally expected a positive but weaker relationship between hoarding and well‐being, an increased likelihood of hoarding was associated with more negative affective balance (*b* = −0.06, 95% CI [−0.09, −0.02]).

Fourth, culture moderated the effects of threat perception on prosocial acts (Δ*R* ^2^ = .02, *F*[3, 894] = 5.93, *p* < .001). Specifically, the effects of threat perception on prosocial acts were statistically significant in the U.K. (*b* = 0.76, 95% CI [0.53, 0.99]), the U.S. (*b* = 0.28, 95% CI [0.07, 0.49]), Germany (*b* = 0.31, 95% CI [0.00, 0.62]), but not in Hong Kong (*b* = 0.02, 95% CI [−0.27, 0.31]). Contrary to H4, the effect of threat perception on prosocial acts was stronger among individualistic cultures (U.K., U.S.) than collectivistic cultures (Hong Kong). Furthermore, culture did not moderate the relationship between threat perception and hoarding (Δ*R* ^2^ = .00, *F*[3, 894] = 0.25, *p* = .860). H5 was not supported.

Finally, culture moderated the effects of benevolent moral identity on prosocial acts (Δ*R* ^2^ = .01, *F*[3, 891] = 5.25, *p* = .001). Specifically, the effects of benevolent moral identity on prosocial acts were stronger in the U.K. (*b* = 0.67, 95% CI [0.49, 0.86]) and the U.S. (*b* = 0.73, 95% CI [0.59, 0.88]) than in Germany (*b* = 0.43, 95% CI [0.25, 0.61]) and Hong Kong (*b* = 0.37, 95% CI [0.14, 0.58]), supporting H6. However, there was no significant relationship between benevolent moral identity and hoarding (*b* = −0.01, 95% CI [−0.18, 0.15]), and culture did not moderate this effect (Δ*R* ^2^ = .00, *F*[3, 891] = 0.17, *p* = .917). H7 was not supported.

Table 2 summarises the indirect effects of threat perception and benevolent moral identity on flourishing and affective balance through prosocial acts or hoarding. In the U.K. and the U.S., threat perception was associated with more prosocial acts, and higher levels of flourishing and more positive affective balance. Meanwhile, threat perception was also associated with a greater likelihood of hoarding but related to more negative affective balance. These indirect effects were reduced in Germany and were not statistically significant in Hong Kong. Across all cultures, benevolent moral identity was associated with more prosocial acts and higher levels of flourishing.

**TABLE 2:** Indirect effect estimates and their corresponding 95% confidence intervals in the U.K., the U.S., Germany, and Hong Kong

Indirect effects	U.K.	U.S.	Germany	Hong Kong
Threat → Prosocial → Flourish	0.20* [0.13, 0.27]	0.07* [0.00, 0.14]	0.08 [−0.01, 0.16]	0.00 [−0.05, 0.06]
Threat → Prosocial → Affect	0.08* [0.04, 0.12]	0.03* [0.00, 0.06]	0.03 [−0.00, 0.07]	0.00 [−0.02, 0.02]
Threat → Hoarding → Flourish	−0.01 [−0.04, 0.00]	−0.01 [−0.04, 0.00]	−0.02 [−0.05, 0.00]	−0.01 [−0.03, 0.01]
Threat → Hoarding → Affect	−0.02* [−0.05, −0.00]	−0.02* [−0.05, −0.00]	−0.03* [−0.06, −0.01]	−0.01 [−0.04, 0.01]
				
MIB → Prosocial → Flourish	0.11* [0.06, 0.16]	0.12* [0.07, 0.16]	0.07* [0.04, 0.11]	0.06* [0.03, 0.10]
MIB → Prosocial → Affect	0.00 [−0.03, 0.04]	0.00 [−0.04, 0.04]	0.00 [−0.02, 0.02]	0.00 [−0.02, 0.02]
MIB → Hoarding → Flourish	0.00 [−0.01, 0.01]	0.00 [−0.01, 0.01]	−0.00 [−0.01, 0.01]	−0.00 [−0.01, 0.01]
MIB → Hoarding → Affect	0.00 [−0.02, 0.03]	0.00 [−0.01, 0.02]	−0.00 [−0.02, 0.02]	−0.00 [−0.03, 0.03]

*Note: n*_UK_ = 251. *n*_US_ = 268. *n*_DE_ = 197. *n*_HK_ = 200. MIB = benevolent moral identity.

^*^ *p* < .05.

## 5 DISCUSSION

We employed the stress and coping framework to understand the emergence and the well‐being outcomes of prosocial acts (donation to/caring for those in need) and hoarding amid the COVID‐19 pandemic. We extended this model by taking benevolent moral identity and cultural differences into account. Our findings partially supported our theory‐driven hypotheses. First, threat perception was a universal ‘pushing’ situational factor that was associated with more prosocial and hoarding behaviours, whereas benevolent moral identity was a ‘pulling’ dispositional factor that promoted prosocial acts during the pandemic. Second, although both prosocial acts and hoarding may act as coping strategies against COVID‐19‐induced stress and anxiety, only the former was associated with favourable, adaptive psychological outcomes. Third, people in more individualistic cultures, such as the U.S. and U.K., were more likely to act prosocially when they perceived the pandemic as more threatening and when they perceived themselves as a moral (benevolent) person. The effects of threat perception and benevolent moral identity were weaker in less individualistic cultures like Germany and Hong Kong.

The findings suggest that amid a global crisis like the COVID‐19 pandemic, acting prosocially may be a viable way to maintain psychological well‐being. This is consistent with the literature on prosociality and well‐being in normal circumstances (Anderson et al., 2014). Prosocial actions increase a sense of connectedness and relatedness, one of the most fundamental psychological needs (Baumeister & Leary, 1995). Particularly during the COVID‐19 when social distancing rules and massive quarantine regulations are in place, being prosocial may serve as a buffer against loneliness induced by the reduction of human interactions (Tse et al., 2020). Given the many psychological and physiological issues associated with loneliness such as depression and weakened immune responses (Hawkley & Cacioppo, 2010), prosocial actions can provide not only altruistic benefits to the community but also personal ‘surplus’ of better well‐being and health to cope with the deadly coronavirus.

Conversely, our findings reveal that buying and stockpiling items in panic does not benefit, but indeed harms psychological well‐being amid the pandemic. Due to its cross‐sectional nature, this study cannot eliminate the alternative explanation that negative affect may lead to greater hoarding tendencies. Nevertheless, the literature suggests that severe cases of hoarding (hoarding disorder) are associated with poorer physical and psychological health, as well as higher risks of other mental health conditions (Kress et al., 2016; Tolin et al., 2019). While acute hoarding behaviour in the pandemic should not be considered as pathological, psychologists and health professionals should monitor closely whether such a buying pattern spills over or turns into prolonged impulsion and obsession of acquiring and retaining objects that hinders daily functioning. It is also important to explore alternative methods, such as telemedicine and online psychotherapies (Tran et al., 2020; Zhang & Ho, 2017), to help those in need especially when social distancing and lockdown measures are in effect.

Culture also plays an important role in predicting prosocial or hoarding behaviour. Surprisingly, threat perception had a stronger positive effect on prosocial acts in individualistic than collectivistic cultures. The bystander model may partly explain the weaker prosocial intentions in response to a crisis among people in collectivistic cultures (Anker & Feeley, 2011). According to the model, noticing an emergency and recognising that someone needs help are the first two steps of helping. However, the acceptance of helping responsibility determines whether a person will implement prosocial acts. Paradoxically, whereas people in collectivistic (vs. individualistic) cultures are more likely to value the welfare of the greater group, they also have greater expectations that someone else in the group will offer help, resulting in a stronger perception of diffusion of responsibility and a reduction of the likelihood of helping (Waldman, Atwater, & Davidson, 2004). This is also consistent with the observation that more individualistic (vs. collectivistic) states in the U.S. are likely to witness more charitable donations and volunteering (Kemmelmeier, Jambor, & Letner, 2006). Furthermore, theorists have also suggested that group or cultural level individualism is associated with a greater extent of perceived personal responsibility and prosociality towards strangers and less intimate in‐groups (Waldman et al., 2004). That said, we are aware that people in Hong Kong were the only cultural group in this study that had experienced the SARS epidemic in 2003, which may have potentially engendered different behavioural patterns. Future research can explore the dynamics between person‐ and group‐level individualism and their relations to altruism while taking into consideration people's previous history of infectious diseases.

### 5.1 Limitations and future directions

Our findings provide preliminary support that prosocial acts, but not hoarding, are viable pathways to maintain good psychological well‐being even under the COVID‐19 pandemic. Nonetheless, readers should interpret our findings with caution, while future research may further delve into this topic. First, the study employed a one‐time survey design that inherently forbade causality inferences. Although previous studies have found similar relationships between prosocial behaviour and well‐being, it is also theoretically possible that positive affect may lead to greater prosociality, as shown by experiments in which affect is manipulated and prosociality observed afterwards (see Lyubomirsky, King, & Diener, 2005 for a review). Likewise, a more negative affective balance (non‐adaptive outcomes) can also feedback to situational appraisals and eventually lead to a greater tendency to hoard food and supplies (Lazarus & Folkman, 1984). Although our theoretical arguments point towards the hypothesised direction of these relationships, future research may employ experimental designs to test causality empirically.

Second, we measured the likelihood of prosocial acts and hoarding in this study instead of the actual counts. Although prosocial tendencies and behaviour are positively correlated, there are occasions when the two depart from one another, such as when people lack the knowledge or resources to offer help (Anker & Feeley, 2011). Future studies may attempt to replicate the findings using alternative designs (e.g., longitudinal follow‐up, lab‐based experiments) and operationalisations (e.g., resource allocation tasks; Piff, Kraus, Côté, Cheng, & Keltner, 2010) to eliminate these potential confounds.

Furthermore, due to the unanticipated, fast development of the COVID‐19 pandemic, we decided to keep all study materials in English, such that we could collect data in different cultures at the beginning of its global outbreak. This goal was achievable due to the relatively high percentages of English speakers in Germany (56%) and Hong Kong (46%). However, these samples should not be considered representative of their corresponding populations. Future studies may collect data from more countries and in their corresponding languages.

Despite these limitations, this study makes several theoretical and practical contributions. First, we demonstrated the utility of integrating situational (threat perception), dispositional (moral identity), and contextual (culture) factors to explain prosociality and hoarding behaviours and their relations to adaptive psychological outcomes during the COVID‐19 crisis. The findings suggest that popular media's attribution of food and supplies hoarding to ‘irrationality’ is likely an oversimplification of this complex social phenomenon and, to some extent, blaming the victims who are coping with the stress and anxiety induced by the pandemic. It is important to ensure the public is well‐informed during the pandemic to reduce threat perception, and thus minimise the likelihood of massive hoarding behaviour (Chu et al., 2021).

Second, our findings underscore the importance of cultural contexts in public health decision‐making. The COVID‐19 is an unprecedented global crisis, and without established procedures, many countries expect international organisations to provide guidelines and solutions that can effectively control the pandemic. Nonetheless, the year 2020 has witnessed a lot of backlashes from people in different countries defying public health regulations such as social distancing or city/state lockdown, illuminating the challenges of implementing ‘one‐size‐fits‐all’ policies that guarantee people's compliance in every culture (BBC, 2020b). For example, preliminary evidence shows that while the majority of people in collectivistic cultures respond positively towards face covering mandates, they perceive social distancing measures less positively (Nguyen et al., 2020). Across cultures, greater threat perception appears to be a double‐edged sword, given its association with both more prosocial acts and hoarding behaviours, but only the former is associated with adaptive psychological outcomes. In turn, political leaders' attempts to downplay the severity of the pandemic may also undermine prosocial behaviours as an adaptive coping strategy. Given our findings, government officials and public health professionals should consider cultural contexts to maximise the effectiveness of the policies and people's compliance. United we stand, divided we fall. Amid the COVID‐19 pandemic, standing by with others in need and being prosocial appears to be one of the adaptive ways to cope with anxiety and stress, and by doing so we stand a greater chance to win this battle.

## ACKNOWLEDGEMENT

This research is partially funded by a General Research Fund (Ref No. 14621920) by the Research Grant Council of Hong Kong SAR government awarded to Y. Hong.

## Supporting information


**Appendix S1:** Supporting information

## Data Availability

The data that support the findings of this study are openly available in the Open Science Framework at https://osf.io/qf42h
