# Supplementary material for: Prosociality and hoarding amid the COVID‐19 pandemic: A tale of four countries
Source: J Community Appl Soc Psychol. 2021 Apr 5;32(3):507–20. doi: 10.1002/casp.2516 (PMC8251234; doi:10.1002/casp.2516)
Supplement: Supplementary file 1 — Appendix S1: Supporting information [file CASP-32-507-s001.docx]

| **Table S1**  *Exploratory Factor Analysis on Items Measuring Perception of COVID-19 as a Threat.* | |
| --- | --- |
| Items | Threat perception |
| worry that COVID-19 would continue to spread in the UK? | .845 |
| worry that you and your loved ones would catch COVID-19? | .744 |
| worry about your daily life being affected by COVID-19? | .703 |
| worry that COVID-19 would harm the social welfare in the UK? | .655 |
| believe that controlling COVID-19 is the highest priority for the UK right now? | .522 |
| worry that COVID-19 would harm the economy in the UK? | .472 |
| *Notes*. *N* = 916. Factors were extracted by principal axis factoring with oblimin rotation. | |

| **Table S2**  *Exploratory Factor Analysis on Prosocial and Hoarding Scale Items.* | | |
| --- | --- | --- |
| Items | Prosocial | Hoarding |
| Donate food to the needy | .961 |  |
| Donate cleaning supplies to the needy | .956 |  |
| Donate supplies to the needy | .939 |  |
| Donate hand sanitizer to the needy | .908 |  |
| Reach out (virtually) to individuals who might feel isolated at this time | .529 |  |
| Educate the elderly in your family about COVID-19 | .467 |  |
| Stock up on food (buy more than you need) |  | .946 |
| Stock up on cleaning supplies (buy more than you need) |  | .899 |
| Stock up on toilet paper (buy more than you need) |  | .875 |
| *Notes*. *N* = 916. Factors were extracted by principal axis factoring with oblimin rotation. | | |

**Table S3**

*R^2^-change and their Corresponding F-statistics for Indirect Effect Models with All Regression Paths Freely Varying across Countries.*

| Parameters | Outcome variables | | | | |
| --- | --- | --- | --- | --- | --- |
|  | Flourishing | |  | Affective balance | |
|  | Δ*R*^2^ | *F* |  | ΔR^2^ | *F* |
| Threat×Culture (a) | .018 | 6.01*** |  | .018 | 6.01*** |
| Prosocial×Culture (b) | .002 | 0.64 |  | .003 | 1.06 |
| Hoarding×Culture (b) | .002 | 0.69 |  | .001 | 0.27 |
| Threat×Culture (c’) | .004 | 1.44 |  | .005 | 2.10 |
|  |  |  |  |  |  |
| MIB×Culture (a) | .014 | 5.11** |  | .014 | 5.11** |
| Prosocial×Culture (b) | .005 | 2.18 |  | .005 | 1.82 |
| Hoarding×Culture (b) | .001 | 0.39 |  | .001 | 0.43 |
| MIB×Culture (c’) | .002 | 0.68 |  | .001 | 0.35 |

*Notes*. *N* = 916. MIB = benevolent moral identity. Degrees of freedom for the *F*-tests were 3 (*df_1_*) and 883 (*df_2_*), respectively.

* *p* < .05. ** *p* < .01. *** *p* < .001.

| **Table S4**  *Correlation matrix of variables.* | | | | | | | | | | | | | | | | | | |
| --- | --- | --- | --- | --- | --- | --- | --- | --- | --- | --- | --- | --- | --- | --- | --- | --- | --- | --- |
| Variables | 1 | 2 | 3 | 4 | 5 | 6 | 7 | 8 | 9 | 10 | 11 | 12 | 13 | 14 | 15 | 16 | 17 | 18 |
| 1. Prosocial | ― |  |  |  |  |  |  |  |  |  |  |  |  |  |  |  |  |  |
| 2. Hoarding | .23^***^ | ― |  |  |  |  |  |  |  |  |  |  |  |  |  |  |  |  |
| 3. Flourishing | .31^***^ | -.02 | ― |  |  |  |  |  |  |  |  |  |  |  |  |  |  |  |
| 4. Affect | .04 | -.16^***^ | .64^***^ | ― |  |  |  |  |  |  |  |  |  |  |  |  |  |  |
| 5. Threat perception | .22^***^ | .20^***^ | -.08^*^ | -.39^***^ | ― |  |  |  |  |  |  |  |  |  |  |  |  |  |
| 6. MI Benevolence | .35^***^ | -.03 | .41^***^ | .25^***^ | .20^***^ | ― |  |  |  |  |  |  |  |  |  |  |  |  |
| 7. MI Justice | .20^***^ | -.02 | .35^***^ | .23^***^ | .14^***^ | .74^***^ | ― |  |  |  |  |  |  |  |  |  |  |  |
| 8. MI Obligation | .17^***^ | -.02 | .37^***^ | .24^***^ | .19^***^ | .73^***^ | .78^***^ | ― |  |  |  |  |  |  |  |  |  |  |
| 9. MI Integrity | .15^***^ | -.05 | .32^***^ | .25^***^ | .14^***^ | .74^***^ | .78^***^ | .83^***^ | ― |  |  |  |  |  |  |  |  |  |
| 10. Age | -.06 | -.12^***^ | .13^***^ | .18^***^ | -.04 | .16^***^ | .19^***^ | .20^***^ | .20^***^ | ― |  |  |  |  |  |  |  |  |
| 11. Sex | .11^**^ | -.04 | .00 | -.11^**^ | .15^***^ | .20^***^ | .10^**^ | .10^**^ | .10^**^ | -.08^*^ | ― |  |  |  |  |  |  |  |
| 12. Subjective SES | .05 | -.02 | .29^***^ | .24^***^ | -.14^***^ | .02 | .03 | .00 | -.02 | .16^***^ | -.14^***^ | ― |  |  |  |  |  |  |
| 13. Education | -.01 | .00 | .01 | -.05 | -.08^*^ | -.11^**^ | -.06 | -.11^**^ | -.15^***^ | -.16^***^ | -.06 | .28^***^ | ― |  |  |  |  |  |
| 14. Liberal | .04 | .04 | -.13^***^ | -.21^***^ | .13^***^ | -.08^*^ | -.10^**^ | -.16^***^ | -.14^***^ | -.23^***^ | .07^*^ | -.06 | .18^***^ | ― |  |  |  |  |
| 15. Conservative | .02 | .07^*^ | .15^***^ | .20^***^ | -.12^***^ | .04 | .03 | .07^*^ | .06 | .15^***^ | -.11^**^ | .16^***^ | -.04 | -.62^***^ | ― |  |  |  |
| 16. US | .10^**^ | .20^***^ | .08^*^ | .04 | .09^**^ | .08^*^ | .14^***^ | .16^***^ | .10^**^ | -.29^***^ | .01 | -.24^***^ | .03 | -.04 | .02 | ― |  |  |
| 17. Hong Kong | -.03 | .11^**^ | -.09^**^ | -.20^***^ | .09^*^ | -.18^***^ | -.24^***^ | -.30^***^ | -.28^***^ | -.17^***^ | .01 | .07^*^ | .04 | .16^***^ | -.01 | -.34^***^ | ― |  |
| 18. UK | .00 | -.06 | .02 | .12^***^ | .08^*^ | .24^***^ | .23^***^ | .30^***^ | .31^***^ | .43^***^ | -.02 | .02 | -.31^***^ | -.20^***^ | .06 | -.40^***^ | -.33^***^ | ― |
| 19. Germany | -.08^*^ | -.27^***^ | -.01 | .03 | -.27^***^ | -.17^***^ | -.16^***^ | -.19^***^ | -.16^***^ | .02 | .00 | .18^***^ | .25^***^ | .10^**^ | -.06 | -.34^***^ | -.28^***^ | -.32^***^ |
| *Note*. *N* = 916. MI = moral identity. SES = socioeconomic status. * *p* < .05. ** *p* < .01. *** *p* < .001. | | | | | | | | | | | | | | | | | | |
